# Supplementary material for: Patterns of Intron Gain and Loss in Fungi
Source: PLoS Biol. 2004 Nov 30;2(12):e422. doi: 10.1371/journal.pbio.0020422 (PMC532390; doi:10.1371/journal.pbio.0020422)
Supplement: Table S1 — Also available at http://genes.mit.edu/NielsenEtAl/. (4.3 MB ZIP). [file pbio.0020422.st001.zip › NielsenEtAl/html/1295.html]

AN4667.1.NCU02464.1.MG03087.1.FG09421.1


```
 CLUSTAL W (1.82) Multiple Sequence Alignments - Introns Inserted


Sequence 1: NCU02464.1	368 aa
Sequence 2: MG03087.1	383 aa
Sequence 3: FG09421.1	408 aa
Sequence 4: AN4667.1	380 aa
Alignment Length: 417 aa
Number Identitical Residues: 277 aa
Alignment Score (without introns) 11423


MG03087.1 	------------------------MSFPA0KMMRRKKNVKKGIQFCLMVCGASGTG1RTT
NCU02464.1	-------------------MFTNTISSRQ~KMMRRKKNVKKGIQFCLMVCGASGTG~---
FG09421.1 	MAKPLEQPKSNTTRILLSWFLASFNIFPL0KMIRRKKNVKKGIQFCLMVCGASGTG1RTT
AN4667.1  	----------------------MSSAYNP0IKLRRKKNVKKGIQFCLMVCGASGTG1RTT
          	                                :***********************  ::

MG03087.1 	FVNTLCGKTVLDHKDSDDPSSAHVEEGVKIKDITVE1LELDEDGTRISLTVVDTPGFGDQ
NCU02464.1	--------------ESFQPPTS----------LSRQ1LELDEEGTRIALTIVDTPGFGDQ
FG09421.1 	FVNTLCGKSVLEHKESDDASGAHVEDGVKIKPITVE1LELDEEGTRISLTIVDTPGFGDQ
AN4667.1  	FVNTLCGKQVLQGKDADDATNAHLEEGVRIKPVTVE1LELDDEGTRISLTIVDTPGFGDQ
          	  .: ...   . .:: :.. :  ...   . :: : ****::****:**:*********

MG03087.1 	VDNEAS2FSEIVG-YLE~REYDNILAEESRIKRNPRFRDNRVHAMLYFITPTGHG2LREL
NCU02464.1	IDNEAS~FSEIVG-YLE~RQYDDILAEESRIKRNPRFRDNRVHAMLYFITPTGHG2LREL
FG09421.1 	IDNEAS~FSEIVG-YLE~RQYDDILAEESRIKRNPRFRDNRVHAMLYFITPTGHG2LREL
AN4667.1  	IDNEAR~HVVTVNKIWE1RQYDDILAEESRIKRNPRFRDNRVHVLLYFITPTGHG~LREL
          	:****  .   *..  * *:**:********************.:********** ****

MG03087.1 	DIELMKRLAPRVNVIPVIGRADSLTPAELAESKKLVMEDIEHYRIPVYNFPYDIEEDDED
NCU02464.1	DIELMKRLAPRVNVIPVIGRADTLTPAELAESKKLVMEDIEHYRIPVYNFPYDIEEDDED
FG09421.1 	DIELMKRLAPRVNVIPVIGRADSLTPAELAESKKLVMEDIEHYRIPVYNFPYDIEEDDED
AN4667.1  	DIELMKRLSPRVNVIPVIGKADSLTPAELAESKKLIMEDIEHYRIPVYNFPYDIEEDDED
          	********:**********:**:************:************************

MG03087.1 	TVEENAELRGLMPFAIVGSEDIIEIGGRKVRARQYPWGVVEVDNPRHSDFLAIRSALLHS
NCU02464.1	TVEENAELRGLMPFAIVGSEDIIEIGGRKVRARQYPWGVVEVDNPRHSDFLAIRSALLHS
FG09421.1 	TVEENAELRGLMPFAIVGSEDVVEIGGRKVRARHYPWGVVEVDNPRHSDFLAIRSALLHS
AN4667.1  	TVEENAELRGLMPFAIVGSDDFVEIDGRKVRARQYPWGVVEVENPRHSDFLAIRSALLHS
          	*******************:*.:**.*******:********:*****************

MG03087.1 	HLADLKEITHDFLYENYRTEALSRSVDGGAG~-~-V2DSSMKPEDLASQSVRLKEEQLRR
NCU02464.1	HLADLKEITHDFLYENYRTEKLSKSVEGGAG~-~-V2DSSMNPEDLASQSVRLKEEQLRR
FG09421.1 	HLADLKEITHDFLYENYRTEKLSKTVDGASG~N2-A~DSSMNPEDLASQSVRLKEEQLRR
AN4667.1  	HLADLKEITHDFLYENYRTEKLSKSVDGAAT2T~GT~DSSINPEDLATQSVRLKEEQLRR
          	******************** **::*:*.:  . .. ***::*****:************

MG03087.1 	EEEKLREIEIKVQREINEKRQELLARESQLREIEARMQREAASSVGP------RDGSEAN
NCU02464.1	EEEKLREIEVKVQREINEKRQELLARESQLREIEARMQREAAAAAQAGTPTTSHSHQEIN
FG09421.1 	EEEKLREIELKVQREINEKRQELLARESQLREIEARMQREAAAQNAG-------TPEPNG
AN4667.1  	EEEKLREIELKVQREIAEKRQELLARESQLREIEARMAREASQGEAA-------EAEQ--
          	*********:****** ******************** ***:              .   

MG03087.1 	GE-DGN
NCU02464.1	GSPDGN
FG09421.1 	GEEQLN
AN4667.1  	------
          	
```
